# Effects of 0.05% cyclosporine, diquafosol sodium, and perfluorohexyloctane eye drops on ocular surface parameters after cataract surgery in patients with type 2 diabetes: a randomized controlled trial

**DOI:** 10.3389/fmed.2026.1960289

**Published:** 2026-10-29

**Authors:** Han Jiang, Meng Ma, Luolei Wang, Shuangle Li

**Affiliations:** 1 Medical School of Ophthalmology and Optometry, North Sichuan Medical College, Nanchong, Sichuan, China; 2 School of Ophthalmology, Chengdu University of Traditional Chinese Medicine, Chengdu, Sichuan, China; 3 The Second People’s Hospital of Jiangyou City, Jiangyou, Sichuan, China; 4 Department of Ophthalmology, Zigong First People's Hospital, Zigong, Sichuan, China

**Keywords:** cataract surgery, cyclosporine, diquafosol sodium, ocular surface, perfluorohexyloctane, type 2 diabetes mellitus

## Abstract

**Objective:**

To compare the short-term effects of cyclosporine, diquafosol sodium, and perfluorohexyloctane on objective ocular surface parameters after cataract surgery in patients with type 2 diabetes.

**Methods:**

In this prospective randomized trial, 144 patients with type 2 diabetes (144 eyes) were randomly assigned to receive standard postoperative care alone or with adjunctive 0.05% cyclosporine, 3% diquafosol sodium, or perfluorohexyloctane eye drops. Of these, 120 (83.3%) completed the 4-week follow-up and were included in the per-protocol analysis. Tear film lipid layer thickness (LLT), tear break-up time (BUT), tear meniscus height (TMH), tear ferning grade, and Schirmer I test (SIT) values were assessed preoperatively and on postoperative day 1 and at weeks 1, 2, 3, and 4. Baseline glycated hemoglobin (HbA1c), diabetes duration, and insulin use were explored as potential modifiers of treatment effects.

**Results:**

Treatment-by-time interactions were observed for all five outcomes (all *P* < 0.001). After multiplicity adjustment, cyclosporine showed a greater increase in BUT than standard control at week 4 (difference, 5.0 s; adjusted CI, 2.7–7.0). Diquafosol showed greater reductions in tear ferning grade and greater increases in Schirmer I values at weeks 3 and 4. At week 4, the respective differences were −1.0 grade (adjusted CI, −2.5 to −1.0) and 5.0 mm (1.0–7.0). No adjusted LLT or TMH comparison interval excluded zero.

**Conclusions:**

Compared with standard care, 0.05% cyclosporine yields short-term improvements in BUT, and diquafosol sodium produces short-term benefits in the Schirmer I test and tear ferning grade. These findings are limited to objective signs and cannot establish an overall treatment ranking among the three agents.

**Trial registration:**

Chinese Clinical Trial Registry https://www.chictr.org.cn/bin/project/edit?pid=265508, ChiCTR2500099433. Reg date: 2025-03-24.

## 1 Introduction

Cataract is one of the leading causes of blindness worldwide, and phacoemulsification combined with intraocular lens implantation has become the standard surgical treatment for visually significant cataract. Nevertheless, postoperative dry eye is a common complication of cataract surgery, with a reported prevalence ranging from 9.8% to 34% (1, 2). The development of postoperative dry eye is multifactorial and has been associated with prolonged use of topical antibiotics and corticosteroids, ocular surface irregularity at the incision site, which may shorten tear break-up time, reduced conjunctival mucin secretion associated with the incision, damage to the cornea–lacrimal gland reflex leading to reduced tear secretion, postoperative inflammation that destabilizes the tear film, and intraoperative microscope light exposure (3, 4).

Patients with diabetes mellitus (DM) are at particularly high risk of postoperative dry eye. DM is a metabolic disorder characterized by chronic hyperglycemia, and its hallmark pathological feature is multisystem damage secondary to dysregulated glucose and lipid metabolism. It is projected that by 2045 the global number of individuals with DM will reach 783.2 million, accounting for approximately 10% of the adult population (5). DM impairs ocular surface health through multiple pathways: long-term hyperglycemia can induce abnormalities of the corneal epithelial basement membrane, reduce corneal nerve fiber density, weaken epithelial repair capacity, and contribute to meibomian gland dysfunction, altered tear composition, and decreased tear film stability (6, 7). Consequently, diabetic patients often present with baseline ocular surface dysfunction before surgery. When cataract surgery is performed in this vulnerable setting, ocular surface impairment may be further aggravated by the incision-related corneal nerve injury, intraoperative light exposure, physical and chemical irritation from surgical instruments and irrigating solutions, and the toxic effects of topical anesthetics further destabilize the tear film (8). In addition, prolonged postoperative use of preservative-containing antibiotic and steroid eye drops may accelerate the loss of conjunctival goblet cells and aggravate ocular surface damage (9).

Current management of postoperative ocular-surface abnormalities is largely limited to symptomatic supportive therapy such as artificial tears and topical anti-inflammatory agents, whereas precise treatment strategies tailored to the diabetic ocular surface remain inadequately defined. In recent years, several targeted agents addressing distinct pathophysiological components of dry eye have emerged. Cyclosporine eye drops suppress T-cell-mediated ocular surface inflammation; diquafosol sodium stimulates aqueous and mucin secretion through activation of P2Y2 receptors; and perfluorohexyloctane stabilizes the tear film surface by forming a barrier that reduces evaporation and improves lipid-layer function (Figure 1) (10). However, most available studies have focused on a single drug in the general population, and direct comparisons of these three agents in the specific setting of cataract surgery in patients with diabetes are scarce. Therefore, in this prospective randomized controlled study, we compared the efficacy of 0.05% cyclosporine, diquafosol sodium, and perfluorohexyloctane for postoperative ocular surface parameters in diabetic patients undergoing cataract surgery and further explored factors associated with treatment response. This study aimed to provide evidence to support individualized management of postoperative ocular surface parameters in this high-risk population. Importantly, diabetic patients often present with subclinical ocular surface dysfunction before cataract surgery, with reduced tear break-up time, decreased Schirmer values, and MGD. In this context, cataract surgery may exacerbate pre-existing ocular surface dysfunction rather than induce *de novo* dry eye disease. Therefore, the present study was designed to compare the effects of three topical agents on objective ocular surface parameters during the early postoperative period in diabetic patients, rather than to diagnose and treat incident postoperative dry eye disease. In accordance with TFOS DEWS II criteria, which require both symptoms and signs for a formal diagnosis of dry eye disease, we acknowledge that patient-reported outcomes were not collected and thus a definitive diagnosis could not be established postoperatively.

**Figure 1:**
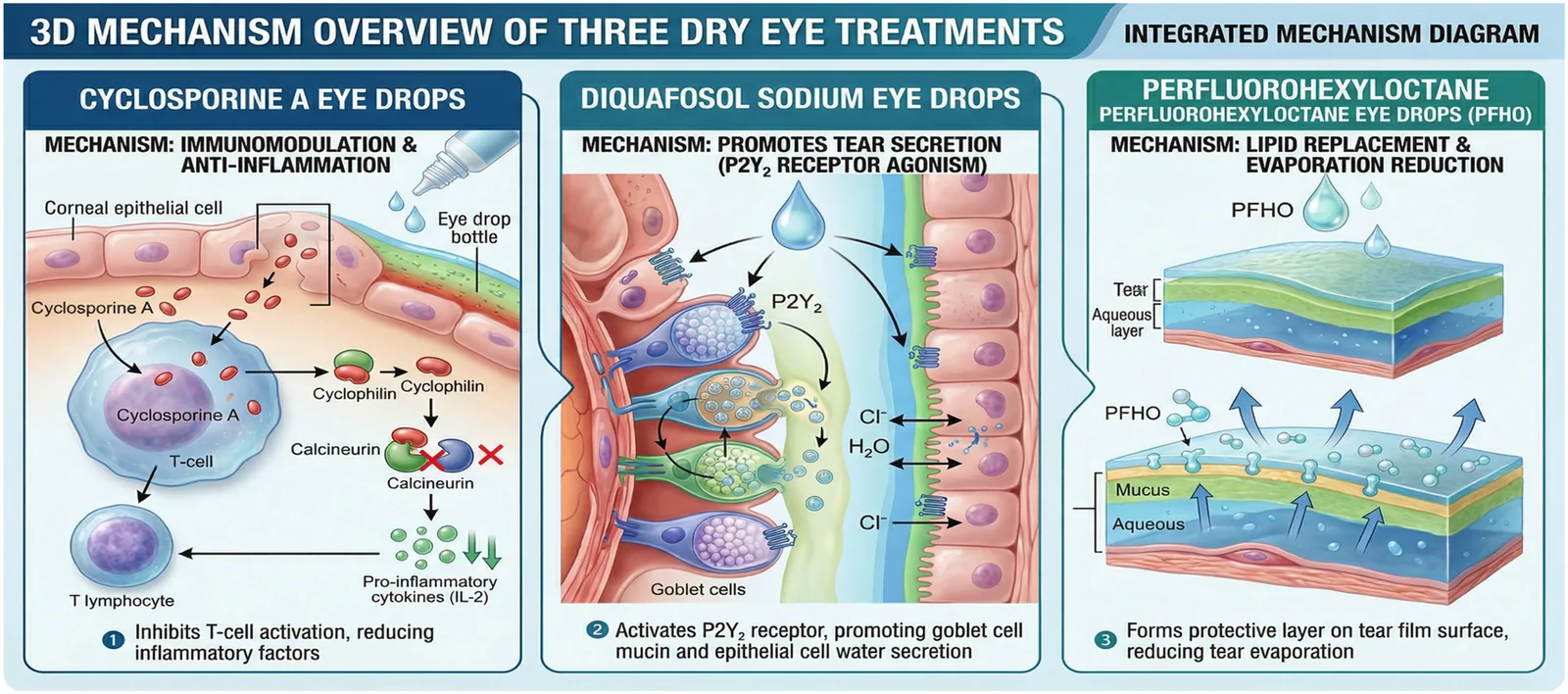
Mechanisms of action of three eye drops.

## 2 Materials and methods

This study adhered to the principles of the Declaration of Helsinki and was approved by the Ethics Committee of The First People's Hospital of Zigong (No. 2024-159). Written informed consent was obtained from all participants. The study was registered with the Chinese Clinical Trial Registry (ChiCTR2500099433).

### 2.1 Study design and participants

This prospective, randomized, parallel-group controlled trial enrolled patients who underwent phacoemulsification with one-stage intraocular lens implantation at our hospital between April 2025 and December 2025.Inclusion criteria were as follows: age 50–75 years; diagnosis of type 2 diabetes mellitus; eligibility for cataract surgery with Emery nuclear grading I–IV; and no obvious preoperative subjective symptoms of dry eye (OSDI score <13 and no ocular discomfort complaints). Exclusion criteria included systemic connective tissue disease or autoimmune disease; conditions known to markedly affect tear secretion or dry eye risk, such as Sjögren syndrome or thyroid-related disease; eyelid or ocular adnexal disorders affecting the ocular surface (e.g., entropion, ectropion, eyelid injury, lagophthalmos, dacryocystitis, or pterygium); active ocular inflammation, allergic ocular surface disease, or contact lens wear within 3 months; coexisting ocular disorders such as ocular trauma, uveitis, elevated intraocular pressure/glaucoma, or keratoconjunctivitis; ocular surgery or laser treatment within 6 months; use of topical medications such as corticosteroids, artificial tears, NSAIDs, or antiglaucoma drugs within 3 months preoperatively; and anticipated need for excessive phaco energy likely to substantially affect the postoperative ocular surface (11). Participants were withdrawn if severe intraoperative or postoperative complications occurred, including corneal epithelial or endothelial injury, retrobulbar or intraocular hemorrhage, postoperative high intraocular pressure, uveitis, persistent corneal edema, or endophthalmitis. Subjects who withdrew consent or failed to complete scheduled follow-up were also considered withdrawn.

### 2.2 Sample size estimation

Sample size was estimated using PASS version 27.0 for a one-way ANOVA with four groups, a two-sided *α* of 0.05, and 75% power. The calculation used Cohen's standardized effect size f = 0.28–0.30, a medium-to-large range under conventional benchmarks (12). Allowing for an anticipated 20% attrition rate (*N*_adjusted = *N*/[1−r]), the planned enrollment was 150 participants.

### 2.3 Data collection and ocular surface assessments

General demographic and clinical data were collected at the initial visit, including age, living environment, past medical history, allergy history, local and systemic medication history, systemic diseases such as Sjögren syndrome, and family history. Diabetes duration, insulin use, and HbA1c were also recorded.

Visual acuity (VA) was assessed using a standard logarithmic visual acuity chart; both uncorrected and corrected VA were recorded. Intraocular pressure (IOP) was measured three times using a non-contact tonometer, and the mean value was used. Lipid layer thickness (LLT) was measured using a LipiView ocular surface interferometer and recorded in interferometric color units (ICU), approximately equivalent to nm. An LLT ≤75 nm was used as an instrument-specific indicator unique to this study, suggesting a relatively thin lipid layer and a potential for meibomian gland dysfunction (MGD). According to current evidence, diagnostic performance at this cutoff value is only moderate, and optimal cutoff values have been reported for each specific device (13). Only measurements with a confidence factor (CF) > 0.7 were accepted. Fluorescein tear break-up time (FBUT/BUT) was measured after instillation of 1% fluorescein sodium or gentle contact with a fluorescein strip moistened without excess topical solution. The interval from the last blink to the appearance of the first dark spot on the cornea was recorded. The mean of three measurements was used. A fluorescein BUT <10 s was considered abnormal according to clinical guidance (14). Tear meniscus height (TMH) was observed under slit-lamp microscopy at the junction of the tear meniscus and lid margin. A TMH ≤0.35 mm was used as a study-specific, suggestive indicator of reduced tear volume, rather than as a stand-alone diagnostic criterion. Recent diagnostic work supports TMH-based assessment while also highlighting the potential value of additional measures, including inter-eye differences (15). Tear ferning test (TFT) was performed without topical anesthesia. Approximately 0.1 μL of tear fluid was collected from the conjunctival sac, placed on a clean glass slide, air-dried at room temperature (20–26 °C, 50% humidity) for 7–10 min, and examined microscopically. Compact, dense fern-like crystallization indicated better tear quality, whereas fragmented or absent crystallization suggested abnormal tear composition (Figure 2) (16, 17).

**Figure 2:**
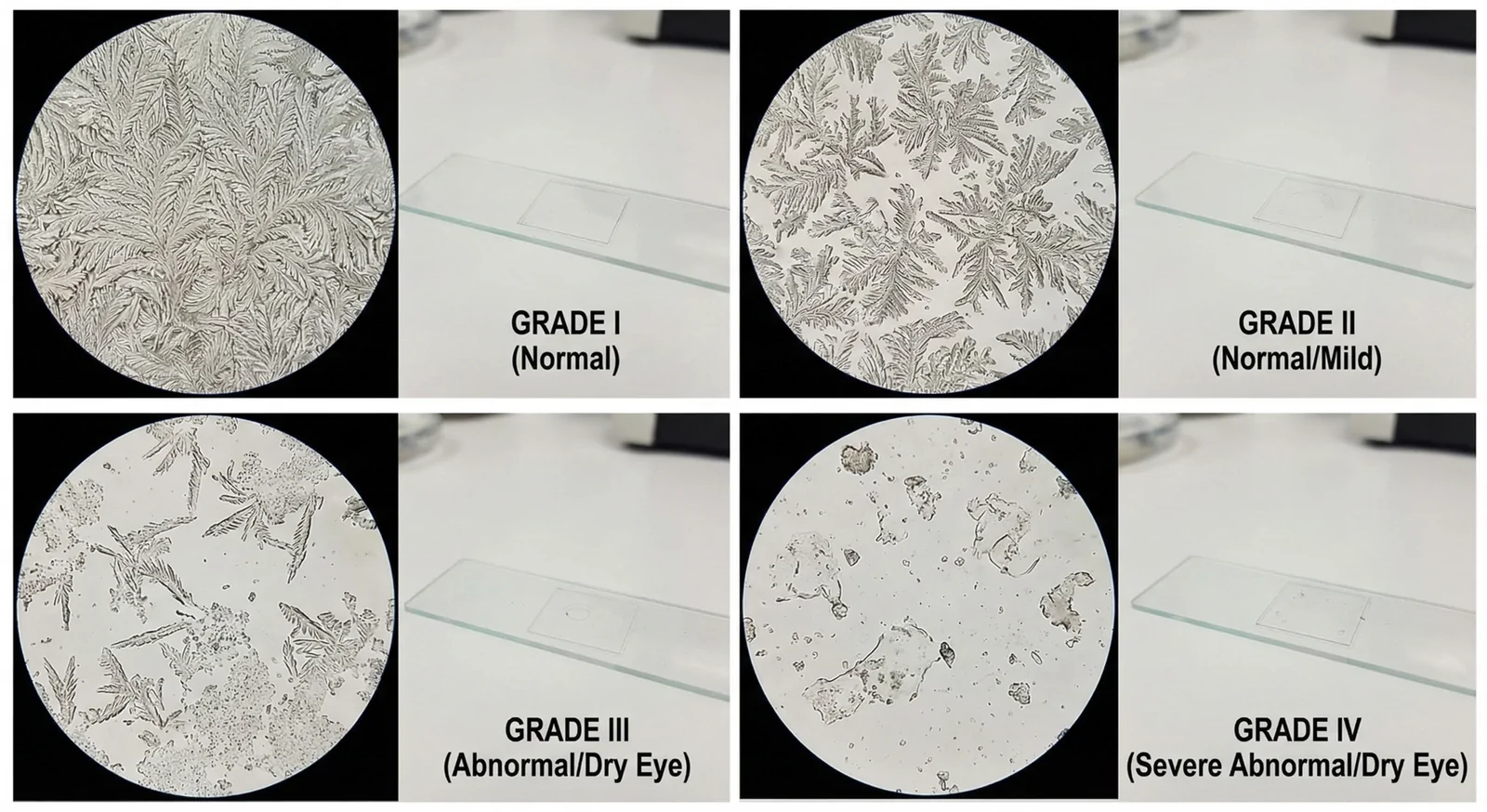
Tear fern test grading(GRADE I: grade I displays uniform and dense fern-like crystallization, with very small gaps between branches; GRADE II: grade II crystallization shows fewer branches, smaller shapes, and increased gaps between branches; GRADE III: grade III crystallization has significantly fewer branches, with larger gaps between them, large enough to form new crystals; GRADE IV: grade IV nearly shows no fern-like crystallization, only a small amount of irregular, indeterminate crystals can be observed.).

Schirmer I test (SIT) was performed without topical anesthesia using standard Schirmer strips (5 mm  ×  35 mm) placed at the junction of the lateral and middle thirds of the lower eyelid margin. The wetted length after 5 min was recorded.

Primary outcome measures: SIT and BUT.

Secondary outcome measures: LLT, TMH, and TFT.

### 2.4 Randomization and treatment groups

A total of 202 type 2 diabetic patients scheduled for cataract surgery were screened. Fifty-eight patients were excluded because they did not meet the inclusion criteria, met exclusion criteria, or declined participation. Ultimately, 144 patients (144 eyes) provided written informed consent and were randomized.

All patients underwent a standardized cataract procedure and received standard postoperative treatment consisting of gatifloxacin eye drops three times daily for 2 weeks, and tobramycin-dexamethasone eye drops plus pranoprofen eye drops three times daily for 4 weeks.

Using a computer-generated random number sequence and sealed-envelope allocation concealment, patients were assigned to one of four groups: Group A (standard control, *n* = 36), standard postoperative treatment only; Group B (0.05% cyclosporine, *n* = 36), standard treatment plus 0.05% cyclosporine ophthalmic solution twice daily for 4 weeks; Group C (3% diquafosol sodium, *n* = 36), standard treatment plus 3% diquafosol sodium ophthalmic solution six times daily for 4 weeks; and Group D (perfluorohexyloctane, *n* = 36), standard treatment plus perfluorohexyloctane eye drops four times daily for 4 weeks. Owing to the clearly different dosing schedules and the absence of placebo-matched bottles, this was an open-label study and no participant-, surgeon-, or assessor-masking was implemented. Details of the study medications and main instruments are shown in Table 1.

**Table 1:** Main study instruments and medications.

Study instruments and medications	Source/regulatory status in this study
Standard logarithmic visual acuity chart	Guangdong Yuehua Medical Equipment Co., Ltd., China
Non-contact tonometer	Canon Inc., Japan
Slit-lamp microscope	Chongqing Kanghuaruiming Technology Co., Ltd., China
Autorefractor/phoropter	Topcon Corporation, Japan
Ophthalmic operating microscope	Carl Zeiss, Germany
Phacoemulsification system	Johnson & Johnson Vision, United States
Tear test filter strips	Tianjin Jingming New Technology Development Co., Ltd., China
Fluorescein sodium ophthalmic test strips	Tianjin Jingming New Technology Development Co., Ltd., China
LipiView ocular surface interferometer	TearScience, Inc., United States
Digital biological microscope	Shandong Xuandu Biotechnology Co., Ltd., China
0.05% cyclosporine ophthalmic solution (II)	Shenyang Xingqi Pharmaceutical Co., Ltd., China; domestically manufactured product used in this study
3% diquafosol sodium ophthalmic solution	Guangdong Zhongsheng Pharmaceutical Co., Ltd., China; domestically manufactured product used in this study
Perfluorohexyloctane eye drops	Chengdu Shendi Pharmaceutical Co., Ltd., China; domestically manufactured product used in this study
Tobramycin-dexamethasone eye drops	s.a. Alcon-Couvreur n.v., Belgium; imported product used in routine postoperative treatment
Gatifloxacin eye drops	Chuxiong Laoboyuntang Pharmaceutical Co., Ltd., China
Pranoprofen eye drops	Guangdong Zhongsheng Pharmaceutical Co., Ltd., China

All study medications were obtained through routine hospital procurement channels. Based on the information provided in Table 1, the three active study drugs used in this trial were manufactured in China, whereas tobramycin-dexamethasone eye drops were an imported product from Belgium.

The planned clinical follow-up period was 1 month postoperatively. During follow-up, 24 patients were lost to follow-up because of long travel distance, self-perceived satisfactory recovery, relocation for work, or other personal reasons. Therefore, 120 patients (120 eyes) completed all visits and were included in the per-protocol analysis: 30 in the cyclosporine group, 30 in the diquafosol sodium group, 30 in the perfluorohexyloctane group, and 30 in the standard control group. This was a per-protocol analysis; no intention-to-treat analysis was performed because post-randomization outcome data were not collected for participants who were lost to follow-up. A CONSORT flow diagram is presented in Figure 3. The high follow-up rate (83.3%) suggests that selection bias due to loss to follow-up is likely limited, though this cannot be formally excluded.

**Figure 3:**
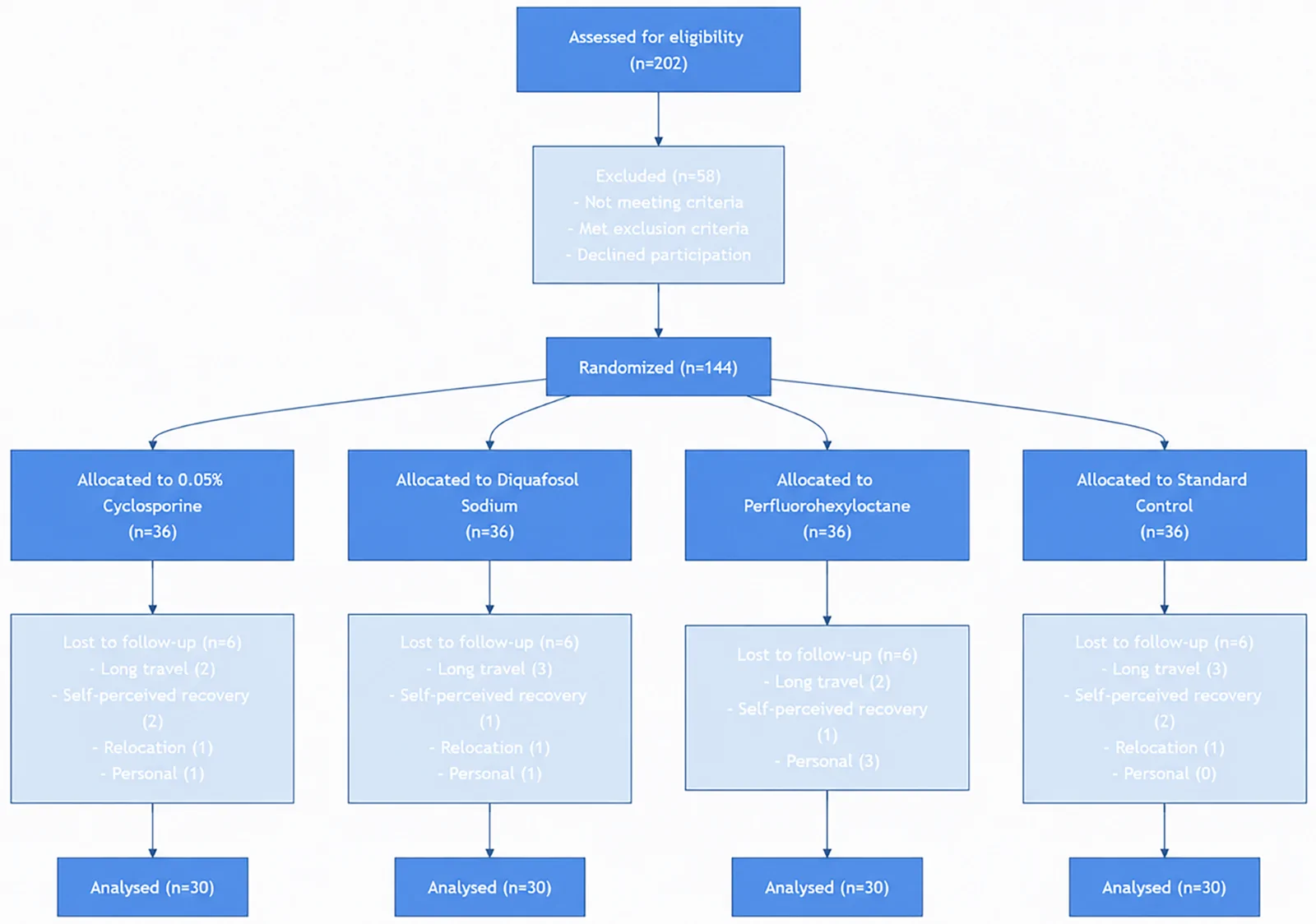
Technical route.

Subgroup analyses were performed according to diabetes duration (<5 years, 5–10 years, and >10 years) (18), HbA1c level (<7.0%, 7.0% to <8.5%, and ≥8.5%) (19, 20), and insulin use (current insulin treatment vs. non-insulin treatment). Baseline values were considered in the analyses. These subgroup analyses were exploratory and were not used as the basis for confirmatory efficacy claims.

### 2.5 Statistical methods

SPSS 27.0 and RStudio software was used. The normality of continuous variables was assessed using the Shapiro–Wilk test. Variables that followed a normal distribution were expressed as mea*n* ± standard deviation, and comparisons between groups were performed using one-way analysis of variance (ANOVA); variables that did not follow a normal distribution were expressed as median (interquartile range), and comparisons between groups were performed using the Kruskal–Wallis H test, with pairwise comparisons conducted using the Dunn test. Categorical data are presented as frequencies (percentages), and comparisons between groups were performed using the *χ*^2^ test or Fisher's exact test. For repeated-measure data, the Friedman test was used to analyze within-group time effects, and pairwise comparisons adjusted with the Bonferroni correction were performed when necessary.

Longitudinal effects were assessed using rank-based F1-LD-F1 analysis, with treatment as the between-subject factor and time as the within-subject factor across baseline and five postoperative visits. ANOVA-type statistics, approximate degrees of freedom, and *P* values for treatment, time, and their interaction are reported in Table 4. These tests assess relative marginal effects. Separately, between-group differences in median individual change from baseline were estimated using 200,000 percentile-bootstrap replicates, resampling patients within treatment groups while preserving complete trajectories. Bonferroni adjustment covered 150 contrasts (six treatment pairs × five outcomes × five visits), yielding 99.9667% intervals targeting 95% familywise coverage, conditional on bootstrap validity. Pointwise 95% intervals are provided in Supplementary Table 3–S4. These original-unit contrasts are distinct from the rank-based model effects.

Exploratory treatment-effect modification at week 4 was assessed separately for each outcome and baseline factor (HbA1c, diabetes duration, or insulin use). For LLT, BUT, TMH, and SIT, linear models included the baseline outcome, treatment, the factor, and their interaction; interaction terms were jointly assessed using HC3-robust Wald *F* tests. HbA1c and duration were continuous, and insulin use was binary. TFT was analyzed using proportional-odds cumulative-logit models adjusted for categorical baseline TFT, with interactions assessed by likelihood-ratio chi-square tests. TFT inferences remain provisional because proportional-odds diagnostics yielded no interpretable results. Holm correction covered the 15 interaction tests separately from the 150 bootstrap contrasts; coefficient intervals were unadjusted. Revision-stage analyses were exploratory, and multiplicity control did not cover all manuscript analyses. Tests were two-sided where applicable, with significance assessed at 0.05 after the specified adjustment.

## 3 Results

Baseline characteristics, including age, sex, diabetes duration, insulin use, HbA1c, and preoperative ocular surface parameters (VA, IOP, LLT, BUT, TMH, tear ferning grade, and SIT), did not differ significantly among the four groups (all *P* > 0.05), indicating good comparability at baseline. No obvious drug-related adverse reactions or serious complications were observed during follow-up (Tables 2, 3). Because the study was open-label and postoperative symptom outcomes were not captured, the efficacy interpretation below focuses on objective signs.

**Table 2:** Comparison of baseline characteristics and ocular surface parameters in patients with diabetes and cataract*.

Variable	0.05% Cyclosporine Group	3% Diquafosol Sodium Group	Perfluorohexyloctane Group	Standard Control Group	*P*	Statistical Test
Age (years)	71.0 (68.0, 73.0)	71.0 (68.0, 73.0)	67.0 (63.0, 72.0)	68.0 (63.0, 72.0)	0.103	Kruskal–Wallis H test
Insulin use	8 (26.7%)	6 (20.0%)	8 (26.7%)	11 (36.7%)	0.546	Chi-square test
Duration of diabetes (years)	10.0 (8.0, 15.0)	10.0 (7.0, 12.0)	8.0 (5.0, 10.0)	9.5 (6.0, 14.0)	0.25	Kruskal–Wallis H test
Preoperative visual acuity (LogMAR)	0.10 (0.04, 0.15)	0.10 (0.06, 0.15)	0.13 (0.08, 0.20)	0.10 (0.02, 0.12)	0.125	Kruskal–Wallis H test
Intraocular pressure (mmHg)	13.7 (12.6, 16.3)	15.1 (13.2, 17.2)	14.4 (13.2, 17.2)	13.7 (11.8, 17.2)	0.644	Kruskal–Wallis H test
Lipid layer thickness (nm)	55.0 (39.0, 76.0)	45.0 (39.0, 69.0)	55.5 (39.0, 79.0)	53.0 (38.0, 70.0)	0.483	Kruskal–Wallis H test
Tear film break-up time (s)	4.0 (3.0, 5.0)	3.0 (2.0, 5.0)	4.5 (3.0, 5.0)	3.5 (2.0, 5.0)	0.598	Kruskal–Wallis H test
Tear meniscus height (mm)	0.20 (0.15, 0.20)	0.19 (0.15, 0.20)	0.20 (0.15, 0.20)	0.20 (0.15, 0.22)	0.191	Kruskal–Wallis H test
Ferning grade	3.0 (2.0, 4.0)	3.0 (3.0, 3.0)	3.0 (3.0, 4.0)	3.0 (2.0, 3.0)	0.382	Kruskal–Wallis H test
Schirmer I test (mm/5 min)	4.5 (3.0, 6.0)	4.5 (3.0, 5.0)	5.00 (3.00 8.00)	5.0 (3.0, 6.0)	0.27	Kruskal–Wallis H test
Glycated hemoglobin (HbA1c)	7.50 (7.20, 8.50)	7.50 (7.20, 8.10)	7.20 (6.90, 7.80)	7.45 (6.90, 8.20)	0.14	Kruskal–Wallis H te

*Continuous variables are expressed as median (25th percentile, 75th percentile) [M (P25, P75)], and between-group comparisons were performed using the Kruskal–Wallis H test. Categorical variables are expressed as number (percentage) [n (%)], and between-group comparisons were performed using the chi-square test (*χ*^2^ test). All *P* values were > 0.05, indicating that the baseline characteristics of the four groups were balanced and comparable.

**Table 3:** Absolute values of ocular surface parameters at each time point in the four groups.

Parameter	Group	Pre-op	Post-op Day 1	Post-op Week 1	Post-op Week 2	Post-op Week 3	Post-op Week 4
Lipid Layer Thickness (LLT)	Cyclosporine	55.00 [40.50, 76.00]	49.00 [40.0, 65.75]	62.00 [45.50, 72.00]	62.00 [46.75, 77.50]	62.00 [45.50, 70.75]	62.00 [50.75, 76.75]
	Diquafosol Sodium	45.00 [39.00, 68.75]	40.00 [39.00, 55.50]	47.50 [41.00, 68.75]	53.00 [39.00, 70.50]	47.50 [39.00, 69.75]	48.00 [41.00, 67.25]
	Perfluorohexyloctane	55.50 [39.50, 77.0]	45.00 [39.00, 65.25]	47.50 [41.25, 67.50]	50.00 [45.00, 70.75]	55.50 [45.50, 70.00]	60.50 [51.25, 86.25]
	Control	53.00 [38.25, 68.00]	43.00 [39.00, 56.50]	57.50 [39.75, 72.75]	57.50 [42.0, 76.75]	58.00 [42.25, 78.25]	58.00 [43.00, 78.75]
Tear Film Break-Up Time (BUT)	Cyclosporine	4.00 [3.00, 5.00]	3.00 [2.00, 3.00]	8.00 [6.25, 10.00]	8.50 [8.00, 10.00]	8.50 [8.00, 10.00]	10.00 [8.00, 10.00]
	Diquafosol Sodium	3.00 [2.00, 5.00]	3.00 [2.00, 4.75]	5.00 [3.25, 5.00]	5.00 [5.00, 6.00]	6.00 [5.00, 6.00]	7.00 [6.00, 8.00]
	Perfluorohexyloctane	4.50 [3.00, 5.00]	3.00 [2.00, 4.00]	3.50 [3.00, 5.00]	5.00 [3.25, 5.00]	4.50 [3.00, 5.00]	5.00 [4.00, 5.00]
	Control	3.50 [2.00, 5.00]	3.00 [2.00, 4.00]	5.00 [4.00, 5.75]	6.00 [5.00, 6.00]	5.00 [5.00, 6.00]	5.00 [4.00, 6.00]
Tear Meniscus Height (TMH)	Cyclosporine	0.20 [0.16, 0.20]	0.20 [0.13, 0.20]	0.20 [0.20, 0.20]	0.20 [0.20, 0.28]	0.20 [0.20, 0.20]	0.20 [0.20, 0.24]
	Diquafosol Sodium l	0.19 [0.15, 0.20]	0.15 [0.10, 0.20]	0.20 [0.15, 0.20]	0.20 [0.20, 0.20]	0.20 [0.20, 0.20]	0.20 [0.20, 0.25]
	Perfluorohexyloctane	0.20 [0.15, 0.20]	0.20 [0.12, 0.20]	0.20 [0.20, 0.20]	0.20 [0.20, 0.20]	0.20 [0.20, 0.25]	0.20 [0.20, 0.25]
	Control	0.20 [0.15, 0.21]	0.15 [0.11, 0.20]	0.20 [0.15, 0.22]	0.20 [0.15, 0.22]	0.20 [0.15, 0.20]	0.20 [0.15, 0.20]
Tear Ferning Grade	Cyclosporine	3.00 [2.25, 4.00]	3.00 [3.00, 4.00]	2.00 [2.00, 3.00]	3.00 [2.50, 3.00]	3.00 [2.00, 3.00]	3.00 [2.00, 3.00]
	Diquafosol Sodium	3.00 [3.00, 3.00]	3.50 [3.00, 4.00]	3.00 [2.00, 3.00]	2.00 [2.00, 3.00]	2.00 [2.00, 2.00]	2.00 [1.00, 2.00]
	Perfluorohexyloctane	3.00 [3.00, 3.75]	4.00 [3.00, 4.00]	3.00 [3.00, 4.00]	3.00 [2.25, 3.00]	3.00 [3.00, 3.00]	3.00 [2.00, 3.00]
	Control	3.00 [2.00, 3.00]	3.00 [3.00, 4.00]	2.00 [2.00, 3.00]	3.00 [3.00, 3.75]	3.00 [3.00, 3.75]	3.00 [3.00, 3.75]
Schirmer I Test	Cyclosporine	4.50 [3.00, 6.00]	3.00 [2.00, 5.00]	5.00 [5.00, 6.00]	6.00 [5.25, 8.00]	6.00 [6.00, 8.00]	8.00 [7.00, 10.00]
	Diquafosol Sodium	4.50 [3.00, 5.00]	3.00 [1.25, 5.00]	7.00 [5.00, 8.00]	8.00 [6.00, 10.00]	10.00 [6.00, 10.00]	10.00 [8.00, 10.75]
	Perfluorohexyloctane	5.00 [3.00, 8.00]	5.00 [3.00, 6.75]	6.00 [5.00, 8.00]	6.00 [5.00, 8.00]	7.00 [6.00, 8.00]	8.00 [6.00, 10.00]
	Control	5.00 [3.00, 6.00]	3.00 [2.00, 5.00]	6.00 [5.00, 8.00]	6.00 [5.00, 8.00]	5.00 [5.00, 6.00]	5.00 [5.00, 6.00]

Treatment-by-time interactions were observed for all five outcomes (Table 4). Following Bonferroni adjustment across 150 comparisons, five active-versus-control intervals excluded zero (Table 5). At week 4, cyclosporine showed a greater median increase in BUT than standard control (difference, 5.0 s; adjusted CI, 2.7 to 7.0). Diquafosol sodium showed greater reductions in tear ferning grade at weeks 3 and 4 (both −1.0 grade; adjusted CIs, −2.0 to −1.0 and −2.5 to −1.0, respectively) and greater increases in Schirmer I test at weeks 3 and 4 (3.0 mm, 0.5 to 5.5; and 5.0 mm, 1.0 to 7.0, respectively). All other active-versus-control adjusted intervals included or touched zero, including every LLT and TMH comparison.

**Table 4:** Rank-based longitudinal tests.

Outcome/effect	ATS	Approximate df	*P* value
LLT/Treatment	1.0232	2.9579	0.3803
LLT/Time	47.7227	3.3434	<0.001
LLT/Treatment × time	3.4362	9.2972	<0.001
BUT/Treatment	32.1071	2.4176	<0.001
BUT/Time	205.1668	3.9161	<0.001
BUT/Treatment × time	24.0564	9.2938	<0.001
TMH/Treatment	0.9725	2.8544	0.4015
TMH/Time	42.0754	3.6275	<0.001
TMH/Treatment × time	4.8131	8.8783	<0.001
Tear ferning/Treatment	7.0014	2.6957	<0.001
Tear ferning/Time	63.1926	3.4844	<0.001
Tear ferning/Treatment × time	16.0673	8.9228	<0.001
Schirmer I/Treatment	3.0990	2.9140	0.0269
Schirmer I/Time	142.7362	3.1703	<0.001
Schirmer I/Treatment × time	10.0581	9.5817	<0.001

LLT, lipid layer thickness; BUT, tear break-up time; TMH, tear meniscus height; ATS, ANOVA-type statistic; df, degrees of freedom. Treatment×time denotes the treatment-by-time interaction. Approximate degrees of freedom are reported.

**Table 5:** All active-treatment versus standard-control comparisons.

Outcome	Visit	CsA−control	DQS−control	PFHO−control
LLT (nm)	D1	2.0 (−4.5, 10.5)	4.0 (−9.0, 11.0)	6.0 (−13.0, 12.0)
	W1	0.0 (−10.0, 8.0)	0.0 (−10.0, 3.0)	0.0 (−18.0, 1.5)
	W2	1.0 (−10.0, 10.0)	−0.5 (−10.0, 5.0)	−2.0 (−13.0, 5.0)
	W3	−4.0 (−10.0, 7.0)	−4.5 (−10.0, 2.0)	−1.5 (−17.3, 4.5)
	W4	0.0 (−9.5, 9.0)	−4.0 (−12.8, 5.0)	1.0 (−7.0, 11.5)
BUT (s)	D1	0.5 (−2.0, 2.0)	1.0 (−0.5, 2.0)	0.0 (−2.0, 2.0)
	W1	3.0 (0.0, 6.0)	0.0 (−2.0, 2.0)	−1.0 (−3.0, 1.0)
	W2	2.5 (0.0, 5.0)	−0.5 (−2.0, 2.0)	−2.5 (−4.0, 0.0)
	W3	3.5 (0.0, 6.0)	2.0 (−2.0, 4.0)	−1.0 (−3.0, 1.0)
	W4	5.0 (2.7, 7.0) *	2.0 (0.0, 5.0)	−1.0 (−2.0, 2.0)
TMH (mm)	D1	0.030 (−0.050, 0.100)	0.030 (−0.050, 0.100)	0.030 (−0.045, 0.100)
	W1	0.000 (0.000, 0.000) †	0.000 (0.000, 0.040)	0.000 (0.000, 0.050)
	W2	0.000 (0.000, 0.080)	0.000 (0.000, 0.050)	0.000 (0.000, 0.050)
	W3	0.000 (0.000, 0.010)	0.035 (0.000, 0.080)	0.000 (0.000, 0.080)
	W4	0.000 (0.000, 0.050)	0.050 (0.000, 0.115)	0.000 (0.000, 0.100)
Tear ferning (grade)	D1	0.0 (−1.0, 1.0)	0.0 (−1.0, 1.0)	0.0 (−1.0, 1.0)
	W1	0.0 (−0.5, 1.0)	−1.0 (−1.0, 1.0)	0.0 (−1.0, 2.0)
	W2	0.0 (−1.5, 0.0)	−1.0 (−2.0, 0.0)	0.0 (−1.0, 0.5)
	W3	0.0 (−1.5, 0.0)	−1.0 (−2.0, −1.0) *	0.0 (−1.0, 0.5)
	W4	0.0 (−1.5, 0.0)	−1.0 (−2.5, −1.0) *	0.0 (−1.0, 0.0)
Schirmer I (mm)	D1	0.0 (−2.0, 2.0)	0.0 (−2.0, 2.0)	1.0 (−2.5, 2.5)
	W1	−0.5 (−2.0, 2.0)	1.5 (−1.0, 3.0)	−1.5 (−2.5, 2.0)
	W2	0.0 (−2.0, 2.0)	1.0 (−0.5, 4.0)	−0.5 (−3.0, 2.5)
	W3	1.0 (−1.0, 4.0)	3.0 (0.5, 5.5) *	1.5 (−2.0, 4.0)
	W4	4.0 (0.0, 5.3)	5.0 (1.0, 7.0) *	3.0 (−0.5, 5.0)

LLT, lipid layer thickness; BUT, tear break-up time; TMH, tear meniscus height; CsA, 0.05% cyclosporine A; DQS, 3% diquafosol sodium; PFHO, perfluorohexyloctane; CI, confidence interval; D1, postoperative day 1; W1–W4, postoperative weeks 1–4; nm, nanometers; s, seconds; mm, millimeters. Values are differences in median individual change from baseline (active treatment minus standard control), with Bonferroni-adjusted CIs in parentheses.

*indicates that the adjusted CI excludes zero; † indicates a zero-width adjusted CI, which does not establish equivalence.

Among active treatments, adjusted BUT intervals favored cyclosporine over diquafosol sodium at weeks 1 and 2 and over perfluorohexyloctane at weeks 1–4 (Supplementary Table 3–S3). At week 4, the cyclosporine-minus-perfluorohexyloctane difference was 6.0 s (adjusted CI, 3.0 to 7.0), whereas the cyclosporine-minus-diquafosol sodium interval included zero (3.0 s; 0.0 to 4.0). No other active-treatment adjusted interval excluded zero. In particular, the week-4 diquafosol sodium -minus-perfluorohexyloctane LLT difference was −5.0 nm (−16.0 to 3.8), which did not support the previously stated LLT superiority after adjustment.

Within-group postoperative changes (descriptive secondary analyses).

### 3.1 Postoperative ocular surface parameters in patients with diabetes mellitus complicated with cataract

All five ocular surface outcomes changed over time within each group (Friedman tests, all *P* < 0.001; Tables 6–8). Descriptively, several parameters worsened immediately after surgery and subsequently recovered. BUT increased most prominently in the cyclosporine group, while the diquafosol sodium group showed reductions in tear ferning grade and increases in Schirmer I values. LLT recovered from its early postoperative decline, whereas changes in median TMH were generally small. These within-group patterns alone do not establish superiority over standard care.

**Table 6:** Overall Friedman Test Results for Ocular Surface Parameters in the Four Groups.

Parameter	Group	Pre-op	Post-op Day 1	Post-op Week 1	Post-op Week 2	Post-op Week 3	Post-op Week 4	*χ* ^2^	df	*P* value
Lipid Layer Thickness (LLT)	0.05% Cyclosporine Group	3.02	1.880	3.70	4.03	3.83	4.53	46.948	5.00	<0.001
	3% Diquafosol Sodium Group	3.08	2.120	3.92	4.13	3.68	4.07	33.895	5.00	<0.001
	Perfluorohexyloctane Group	3.43	1.970	2.67	3.47	4.13	5.33	75.332	5.00	<0.001
	Standard Control Group	3.77	2.150	2.77	3.45	3.98	4.88	57.603	5.00	<0.001
Tear Film Break-Up Time (BUT)	0.05% Cyclosporine Group	1.75	1.250	4.02	4.50	4.42	5.07	129.017	5.00	<0.001
	3% Diquafosol Sodium Group	1.90	1.750	3.13	4.13	4.70	5.38	111.698	5.00	<0.001
	Perfluorohexyloctane Group	3.75	1.900	3.37	3.37	4.28	4.33	52.416	5.00	<0.001
	Standard Control Group	2.90	1.670	3.83	5.28	4.37	2.95	82.966	5.00	<0.001
Tear Meniscus Height (TMH)	0.05% Cyclosporine Group	3.42	2.180	3.82	4.12	3.78	3.68	43.419	5.00	<0.001
	3% Diquafosol Sodium Group	2.80	2.170	3.32	3.50	4.35	4.87	73.992	5.00	<0.001
	Perfluorohexyloctane Group	3.13	2.500	3.62	3.83	3.83	4.08	35.310	5.00	<0.001
	Standard Control Group	3.97	2.320	3.97	3.97	3.55	3.23	39.802	5.00	<0.001
Tear Ferning Grade	0.05% Cyclosporine Group	3.97	4.750	3.45	2.80	3.10	2.93	48.220	5.00	<0.001
	3% Diquafosol Sodium Group	4.98	5.430	3.72	2.95	2.17	1.75	117.685	5.00	<0.001
	Perfluorohexyloctane Group	3.55	4.630	3.75	3.05	3.17	2.85	34.567	5.00	<0.001
	Standard Control Group	3.18	4.430	2.22	3.60	3.78	3.78	50.913	5.00	<0.001
Schirmer I Test	0.05% Cyclosporine Group	2.32	1.370	3.20	4.13	4.55	5.43	115.000	5.00	<0.001
	3% Diquafosol Sodium Group	1.85	1.470	3.47	4.25	4.80	5.17	112.864	5.00	<0.001
	Perfluorohexyloctane Group	2.77	2.050	3.25	3.70	4.25	4.98	62.722	5.00	<0.001
	Standard Control Group	2.77	1.580	4.25	4.82	3.98	3.60	70.000	5.00	<0.001

All comparisons were performed using the Wilcoxon signed-rank test and adjusted using Bonferroni correction for multiple comparisons (15 comparisons). The adjusted significance level was *α*’ = 0.05/15 ≈ 0.0033.

*indicates that the comparison remained statistically significant after the strict Bonferroni correction (*P* < 0.0033).

**Table 7:** Summary of comparisons between each time point and preoperative baseline.

Parameter	Group	Comparison Time Point	Z value	Raw *P* value	Adjusted *P* value
Lipid Layer Thickness (LLT)	Standard Control Group	Post-op Day 1 vs. Pre-op	−3.947	0	<0.001
Tear Film Break-Up Time (BUT)	0.05% Cyclosporine Group	Post-op Week 1 vs. Pre-op	−4.796	<0.001	<0.001
	0.05% Cyclosporine Group	Post-op Week 2 vs. Pre-op	−4.804	<0.001	<0.001
	0.05% Cyclosporine Group	Post-op Week 3 vs. Pre-op	−4.794	<0.001	<0.001
	0.05% Cyclosporine Group	Post-op Week 4 vs. Pre-op	−4.807	<0.001	<0.001
	3% Diquafosol Sodium Group	Post-op Week 1 vs. Pre-op	−3.792	<0.001	<0.001
	3% Diquafosol Sodium Group	Post-op Week 2 vs. Pre-op	−4.513	<0.001	<0.001
	3% Diquafosol Sodium Group	Post-op Week 3 vs. Pre-op	−4.494	<0.001	<0.001
	3% Diquafosol Sodium Group	Post-op Week 4 vs. Pre-op	−4.721	<0.001	<0.001
	Perfluorohexyloctane Group	Post-op Day 1 vs. Pre-op	−3.638	<0.001	<0.001
	Standard Control Group	Post-op Day 1 vs. Pre-op	−3.561	<0.001	<0.001
	Standard Control Group	Post-op Week 2 vs. Pre-op	−4.272	<0.001	<0.001
Tear Meniscus Height (TMH)	Standard Control Group	Post-op Day 1 vs. Pre-op	−3.553	<0.001	<0.001
Tear Ferning Grade	3% Diquafosol Sodium Group	Post-op Week 1 vs. Pre-op	−4.123	<0.001	<0.001
	3% Diquafosol Sodium Group	Post-op Week 2 vs. Pre-op	−4.508	<0.001	<0.001
	3% Diquafosol Sodium Group	Post-op Week 3 vs. Pre-op	−4.795	<0.001	<0.001
	3% Diquafosol Sodium Group	Post-op Week 4 vs. Pre-op	−4.906	<0.001	<0.001
	Standard Control Group	Post-op Day 1 vs. Pre-op	−3.638	<0.001	<0.001
Schirmer I Test	0.05% Cyclosporine Group	Post-op Day 1 vs. Pre-op	−3.704	<0.001	<0.001
	0.05% Cyclosporine Group	Post-op Week 1 vs. Pre-op	−3.552	<0.001	<0.001
	0.05% Cyclosporine Group	Post-op Week 2 vs. Pre-op	−4.230	<0.001	<0.001
	0.05% Cyclosporine Group	Post-op Week 3 vs. Pre-op	−4.325	<0.001	<0.001
	0.05% Cyclosporine Group	Post-op Week 4 vs. Pre-op	−4.558	<0.001	<0.001
	3% Diquafosol Sodium Group	Post-op Week 1 vs. Pre-op	−4.411	<0.001	<0.001
	3% Diquafosol Sodium Group	Post-op Week 2 vs. Pre-op	−4.720	<0.001	<0.001
	3% Diquafosol Sodium Group	Post-op Week 3 vs. Pre-op	−4.718	<0.001	<0.001
	3% Diquafosol Sodium Group	Post-op Week 4 vs. Pre-op	−4.714	<0.001	<0.001
	Perfluorohexyloctane Group	Post-op Week 4 vs. Pre-op	−3.944	<0.001	<0.001
	Standard Control Group	Post-op Day 1 vs. Pre-op	−3.743	<0.001	<0.001
	Standard Control Group	Post-op Week 2 vs. Pre-op	−4.234	<0.001	<0.001

All comparisons were performed using the Wilcoxon signed-rank test and adjusted using Bonferroni correction for multiple comparisons (15 comparisons). The adjusted significance level was *α*' = 0.05/15 ≈ 0.0033.

* indicates that the comparison remained statistically significant after the strict Bonferroni correction (*P* < 0.0033).

**Table 8:** Summary of key comparisons during postoperative recovery.

Parameter	Group	Key Recovery Stage Comparison	Z value	Raw *P* value	Adjusted *P* value
Lipid Layer Thickness (LLT)	0.05% Cyclosporine Group	Post-op Week 1 vs. Post-op Day 1	−3.730	<0.001	<0.001
	0.05% Cyclosporine Group	Post-op Week 2 vs. Post-op Day 1	−3.571	<0.001	<0.001
	0.05% Cyclosporine Group	Post-op Week 4 vs. Post-op Day 1	−3.898	<0.001	<0.001
	3% Diquafosol Sodium Group	Post-op Week 1 vs. Post-op Day 1	−3.982	<0.001	<0.001
	3% Diquafosol Sodium Group	Post-op Week 2 vs. Post-op Day 1	−3.698	<0.001	<0.001
	3% Diquafosol Sodium Group	Post-op Week 4 vs. Post-op Day 1	−3.515	<0.001	<0.001
	Perfluorohexyloctane Group	Post-op Week 2 vs. Post-op Day 1	−3.521	<0.001	<0.001
	Perfluorohexyloctane Group	Post-op Week 3 vs. Post-op Week 1	−3.627	<0.001	<0.001
	Perfluorohexyloctane Group	Post-op Week 4 vs. Post-op Week 2	−3.734	<0.001	<0.001
	Perfluorohexyloctane Group	Post-op Week 4 vs. Post-op Week 3	−3.826	<0.001	<0.001
	Standard Control Group	Post-op Week 1 vs. Post-op Day 1	−4.113	<0.001	<0.001
	Standard Control Group	Post-op Week 3 vs. Post-op Day 1	−4.476	<0.001	<0.001
Tear Film Break-Up Time (BUT)	0.05% Cyclosporine Group	Post-op Week 1 vs. Post-op Day 1	−4.796	<0.001	<0.001
	0.05% Cyclosporine Group	Post-op Week 2 vs. Post-op Day 1	−4.804	<0.001	<0.001
	0.05% Cyclosporine Group	Post-op Week 4 vs. Post-op Day 1	−4.807	<0.001	<0.001
	3% Diquafosol Sodium Group	Post-op Week 1 vs. Post-op Day 1	−4.075	<0.001	<0.001
	3% Diquafosol Sodium Group	Post-op Week 2 vs. Post-op Day 1	−4.520	<0.001	<0.001
	3% Diquafosol Sodium Group	Post-op Week 4 vs. Post-op Day 1	−4.725	<0.001	<0.001
	Perfluorohexyloctane Group	Post-op Week 1 vs. Post-op Day 1	−3.806	<0.001	<0.001
	Perfluorohexyloctane Group	Post-op Week 2 vs. Post-op Day 1	−3.806	<0.001	<0.001
	Standard Control Group	Post-op Week 1 vs. Post-op Day 1	−4.075	<0.001	<0.001
	Standard Control Group	Post-op Week 3 vs. Post-op Day 1	−4.219	<0.001	<0.001
	Standard Control Group	Post-op Week 4 vs. Post-op Week 2	−4.588	<0.001	<0.001
	Standard Control Group	Post-op Week 4 vs. Post-op Week 3	−3.582	<0.001	<0.001
Tear Meniscus Height (TMH)	0.05% Cyclosporine Group	Post-op Week 1 vs. Post-op Day 1	−3.601	<0.001	<0.001
	Standard Control Group	Post-op Week 1 vs. Post-op Day 1	−3.553	<0.001	<0.001
Tear Ferning Grade	0.05% Cyclosporine Group	Post-op Week 1 vs. Post-op Day 1	−3.557	<0.001	<0.001
	3% Diquafosol Sodium Group	Post-op Week 1 vs. Post-op Day 1	−4.245	<0.001	<0.001
	3% Diquafosol Sodium Group	Post-op Week 3 vs. Post-op Week 1	−4.119	<0.001	<0.001
	Standard Control Group	Post-op Week 1 vs. Post-op Day 1	−4.099	<0.001	<0.001
	Standard Control Group	Post-op Week 4 vs. Post-op Week 1	−3.819	<0.001	<0.001
Schirmer I Test	0.05% Cyclosporine Group	Post-op Week 1 vs. Post-op Day 1	−4.321	<0.001	<0.001
	0.05% Cyclosporine Group	Post-op Week 4 vs. Post-op Week 1	−4.414	<0.001	<0.001
	0.05% Cyclosporine Group	Post-op Week 4 vs. Post-op Week 3	−3.587	<0.001	<0.001
	3% Diquafosol Sodium Group	Post-op Week 1 vs. Post-op Day 1	−4.441	<0.001	<0.001
	3% Diquafosol Sodium Group	Post-op Week 2 vs. Post-op Day 1	−4.696	<0.001	<0.001
	Perfluorohexyloctane Group	Post-op Week 1 vs. Post-op Day 1	−3.666	<0.001	<0.001
	Perfluorohexyloctane Group	Post-op Week 4 vs. Post-op Week 1	−3.996	<0.001	<0.001
	Perfluorohexyloctane Group	Post-op Week 4 vs. Post-op Week 2	−3.629	<0.001	<0.001
	Standard Control Group	Post-op Week 1 vs. Post-op Day 1	−4.625	<0.001	<0.001
	Standard Control Group	Post-op Week 2 vs. Post-op Day 1	−4.584	<0.001	<0.001

All comparisons were performed using the Wilcoxon signed-rank test and adjusted using Bonferroni correction for multiple comparisons (15 comparisons). The adjusted significance level was *α*’ = 0.05/15 ≈ 0.0033.

*indicates that the comparison remained statistically significant after the strict Bonferroni correction (*P* < 0.0033).

The F1-LD-F1 analysis identified treatment-by-time interactions for all five outcomes (all *P* < 0.001). In exploratory comparisons adjusted across 150 contrasts, cyclosporine showed a greater median increase in BUT than standard control at week 4 (difference, 5.0 s; adjusted CI, 2.7–7.0). Diquafosol sodium showed greater reductions in tear ferning grade at weeks 3 and 4 (both −1.0 grade; adjusted CIs, −2.0 to −1.0 and −2.5 to −1.0, respectively) and greater increases in Schirmer I values (3.0 mm, 0.5–5.5; and 5.0 mm, 1.0–7.0, respectively). All other active-versus-control adjusted intervals included or touched zero. Among active treatments, BUT comparisons favored cyclosporine over diquafosol sodium at weeks 1–2 and over perfluorohexyloctane at weeks 1–4. These findings remain exploratory, particularly for outcomes with discrete or highly tied values.

### 3.2 Association between baseline factors and treatment response

For LLT, the HbA1c-by-treatment interaction was not statistically significant {HC3 Wald F(3, 111) = 0.891, *P* = 0.448; Holm-adjusted *P* = 1.000}. The remaining continuous-outcome interactions with HbA1c, diabetes duration, or insulin use were also non-significant (Table 9). These analyses do not support selecting a topical treatment on the basis of these baseline factors.

**Table 9:** Exploratory treatment-by-baseline-factor interactions at postoperative week 4.

Baseline factor	Outcome	Test	Statistic	df	Raw P	Holm P
HbA1c	LLT	F	0.891	3, 111	0.448	1
HbA1c	BUT	F	0.525	3, 111	0.666	1
HbA1c	TMH	F	0.81	3, 111	0.491	1
HbA1c	SIT	F	0.394	3, 111	0.758	1
HbA1c	TFT†	χ^2^	0.654	3	0.884	1
Diabetes duration	LLT	F	0.627	3, 111	0.599	1
Diabetes duration	BUT	F	0.665	3, 111	0.575	1
Diabetes duration	TMH	F	0.537	3, 111	0.658	1
Diabetes duration	SIT	F	1.655	3, 111	0.181	1
Diabetes duration	TFT†	χ^2^	1.851	3	0.604	1
Insulin use	LLT	F	0.436	3, 111	0.728	1
Insulin use	BUT	F	0.417	3, 111	0.741	1
Insulin use	TMH	F	0.071	3, 111	0.976	1
Insulin use	SIT	F	1.921	3, 111	0.13	1
Insulin use	TFT†	χ^2^	6.043	3	0.11	1

HbA1c, glycated hemoglobin; LLT, lipid layer thickness; BUT, tear break-up time; TMH, tear meniscus height; SIT, Schirmer I test; TFT, tear ferning test; df, degrees of freedom.

†TFT results remain provisional because proportional-odds diagnostics yielded no interpretable test statistics or *P* values.

In the fitted TFT models, the treatment interactions with HbA1c, diabetes duration, and insulin use yielded likelihood-ratio chi-square statistics of 0.654, 1.851, and 6.043, respectively (each df = 3; *P* = 0.884, 0.604, and 0.110; all Holm-adjusted *P* = 1.000). These results are provisional because proportional-odds diagnostics did not provide interpretable results. At week 4, the diquafosol group had 13 participants with grade I, 16 with grade II, one with grade III, and none with grade IV (Table 10). Grade I was absent from the other groups. Sparse cells persisted after stratification by insulin use (Table 11), limiting model assessment. These distributions are descriptive and do not establish treatment-effect modification.

**Table 10:** Distribution of TFT grades at postoperative week 4.

Treatment	N	Grade I	Grade II	Grade III	Grade IV
Standard control	30	0 (0.0)	5 (16.7)	17 (56.7)	8 (26.7)
Cyclosporine	30	0 (0.0)	13 (43.3)	15 (50.0)	2 (6.7)
Diquafosol	30	13 (43.3)	16 (53.3)	1 (3.3)	0 (0.0)
Perfluorohexyloctane	30	0 (0.0)	10 (33.3)	14 (46.7)	6 (20.0)

Values are *n* (%) within each treatment group. Grades I–IV are ordered from better to worse ferning patterns.

**Table 11:** TFT grade distribution by treatment group and baseline insulin use.

Treatment	Insulin	N	Grade I	Grade II	Grade III	Grade IV
Standard control	No	19	0 (0.0)	3 (15.8)	14 (73.7)	2 (10.5)
Standard control	Yes	11	0 (0.0)	2 (18.2)	3 (27.3)	6 (54.5)
Cyclosporine	No	22	0 (0.0)	7 (31.8)	14 (63.6)	1 (4.5)
Cyclosporine	Yes	8	0 (0.0)	6 (75.0)	1 (12.5)	1 (12.5)
Diquafosol	No	24	11 (45.8)	13 (54.2)	0 (0.0)	0 (0.0)
Diquafosol	Yes	6	2 (33.3)	3 (50.0)	1 (16.7)	0 (0.0)
Perfluorohexyloctane	No	22	0 (0.0)	8 (36.4)	9 (40.9)	5 (22.7)
Perfluorohexyloctane	Yes	8	0 (0.0)	2 (25.0)	5 (62.5)	1 (12.5)

Values are *n* (%) within each treatment-by-insulin subgroup. Insulin use was not randomized. Empty and sparse categories limit ordinal-model diagnostics and precision. This table describes sample composition and does not establish an insulin-related difference in treatment effects; see the overall interaction tests in Table 7.

## 4 Discussion

The participants were restricted to adults aged 50–75 years with type 2 diabetes who were eligible for cataract surgery and had no obvious preoperative dry-eye symptoms. This restriction creates spectrum and selection bias and limits external validity: the results should not be extrapolated to younger adults, adults older than 75 years, people with type 1 diabetes, symptomatic dry eye, or non-surgical populations.

The incidence of postoperative ocular surface imbalance is high among patients with diabetes and cataracts, and this high incidence is the result of the combined effects of metabolic disturbances associated with diabetes and surgical trauma. Previous studies have primarily focused on the prevention and treatment effects on postoperative ocular surface imbalance following standard cataract surgery, while targeted intervention studies addressing postoperative ocular surface parameters in this specific population of patients with diabetes remain relatively limited. This study compared the efficacy of 0.05% cyclosporine, sodium diquafosol, and perfluorohexyloctane eye drops in improving postoperative ocular surface parameters in patients with diabetic cataracts. By systematically evaluating their efficacy using multiple objective ocular surface parameters, the study fills a gap in clinical evidence regarding targeted treatment for postoperative ocular surface parameters in this population.

The F1-LD-F1 analysis identified treatment-by-time interactions for all five objective ocular surface outcomes, indicating different temporal patterns among groups. Exploratory comparisons adjusted across 150 contrasts suggested greater improvement in BUT with cyclosporine than with standard control at week 4, and in Schirmer I test and tear ferning grade with diquafosol sodium than with standard control at weeks 3 and 4.

### 4.1 Standard control group

The standard control group followed the prescribed protocol (mainly including: gatifloxacin eye drops, tobramycin-dexamethasone eye drops, and pranoprofen eye drops), and only received postoperative basic anti-inflammatory and anti-infection treatments.

In the standard control group, LLT, BUT, TMH, and SIT median values were lower on postoperative day 1 than before surgery; the median tear ferning grade remained 3, although its distribution changed. This is closely related to the mechanical transection of corneal nerves caused by surgical trauma, the toxicity of topical anesthetics to the epithelium, and the acute release of inflammatory factors (3). Between 1 and 2 weeks postoperatively, multiple parameters (LLT, BUT, SIT) in this group showed rapid and significant rebound, with some even returning to preoperative levels. This may be related to perioperative corticosteroids controlling inflammation and growth factors promoting epithelial repair. Descriptively, LLT remained approximately stable from weeks 1 to 4; BUT peaked at week 2 and was lower at weeks 3 and 4; SIT was higher at weeks 1–2 than at weeks 3–4; and median TMH returned to approximately its preoperative value. These findings suggest that the interventions in the standard-care group confer a certain capacity for ocular surface repair.

### 4.2 0.05% cyclosporine

In this study, LLT in the 0.05% cyclosporine group improved significantly as early as 1 week postoperatively compared to 1 day postoperatively and remained at a good level at 4 weeks postoperatively. This suggests that 0.05% cyclosporine may, by suppressing T-cell-mediated chronic inflammation of the ocular surface and meibomian glands, fundamentally reduce the impact of inflammatory factors on meibomian gland acini and protect the secretory function of the remaining glands (21). In diabetic patients, the meibomian glands are often in a state of low-grade inflammation, which, in combination with the acute inflammation caused by surgical trauma, exacerbates damage to the ocular surface. 0.05% cyclosporine, a fungal-derived cyclic peptide and calcineurin inhibitor, suppresses T-cell activation, prevents the release of inflammatory cytokines, inhibits inflammation in the conjunctiva and lacrimal glands, and promotes tear secretion. Studies have shown that 0.05% cyclosporine can also increase the density of conjunctival goblet cells and inhibit conjunctival epithelial cell apoptosis (22). In the 0.05% cyclosporine group, BUT was significantly better than preoperative levels starting from 1 week postoperatively, with this improvement being relatively sustained and peaking at 4 weeks postoperatively. The adjusted comparisons suggested greater median improvement in BUT with cyclosporine than with standard control at week 4, with a difference of 5.0 s (adjusted CI, 2.7–7.0). Comparisons also favored cyclosporine over perfluorohexyloctane at weeks 1–4 and over diquafosol sodium at weeks 1–2, but not over diquafosol sodium at week 4. These observations do not directly establish the biological mechanism. In the cyclosporine group, SIT increased from week 1 onward relative to baseline, whereas median TMH showed limited change. However, the adjusted cyclosporine-versus-control SIT and TMH intervals included or touched zero; the observed within-group changes therefore do not establish an additional treatment benefit for these outcomes. The Schirmer I test stimulates conjunctival nerve endings via filter paper, triggering reflex tear secretion that directly affects the aqueous layer (17); however, TMH does not isolate basal tear secretion. Furthermore, studies have shown that topical administration of 0.05% cyclosporine may have a protective effect on the ocular surface (23). Additionally, studies have found that use of 0.05% cyclosporine increases EGF and TGF-*β*1 levels, accelerating corneal epithelial repair and cell proliferation (24). In a randomized controlled trial conducted by Hamada (25) et al. involving 30 patients, those receiving 0.05% cyclosporine eye drops showed more significant improvements in tear osmolarity, TBUT, tear secretion tests, and corneal nerve function one month after cataract surgery compared to those receiving 0.5% carboxymethylcellulose. Similarly, in a randomized fellow-eye study involving 32 patients with newly diagnosed dry eye after cataract surgery, Chung et al. reported improvements in tear film stability and symptoms during 3 months of twice-daily 0.05% cyclosporine treatment, with significantly higher Schirmer I values than in saline-treated fellow eyes at 3 months (26).

### 4.3 Diquafosol sodium

In the diquafosol sodium eye drop group, SIT remained extremely significantly higher postoperatively than preoperatively starting from 1 week post-surgery,with a continuous upward trend at 4 weeks post-surgery,For diabetic patients experiencing postoperative suppression of reflex secretion and a decline in basal lacrimal gland secretory function due to temporary corneal nerve damage (27), sodium diquafosol can directly increase tear volume by promoting the secretion of aqueous and mucin components. Exploratory adjusted comparisons suggested greater reductions in tear ferning grade with diquafosol sodium group than with standard control group at weeks 3 and 4 (both −1.0 grade; adjusted CIs, −2.0 to −1.0 and −2.5 to −1.0, respectively). The tear ferning test is a sensitive indicator of tear electrolyte balance, mucin content, and macromolecular composition (28, 29). Under hyperglycemic conditions, the concentration of mucin 5AC (MUC5AC) drops sharply, whereas diquafosol sodium promotes MUC5AC secretion, forming a hydrophilic gel layer that covers the corneal epithelial surface, locks in moisture, lubricates the ocular surface, and acts as a physical barrier against pathogens, thereby directly optimizing the physicochemical properties of tears and resulting in denser, more regular fern-like crystals (30, 31). As a P2Y2 receptor agonist, diquafosol sodium increases mucin secretion (32) while also promoting the secretion of water and electrolytes by conjunctival epithelial cells, thereby increasing tear volume. It also reduces corneal epithelial cell loss, promotes aqueous flow, and restores the tear film to near-normal levels in terms of both quality and quantity, thereby balancing tear film homeostasis (27, 33). BUT increased within the diquafosol sodium group from week 1 onward. However, all adjusted diquafosol-versus-control BUT intervals included or touched zero. These findings do not establish delayed onset, an optimal treatment duration. For Schirmer I test, exploratory comparisons favored diquafosol sodium group over standard control group at weeks 3 and 4 (differences, 3.0 and 5.0 mm; adjusted CIs, 0.5–5.5 and 1.0–7.0, respectively). Research by Qin (34) et al. further demonstrated that the drug reduces levels of inflammatory mediators such as matrix metalloproteinase-9 (MMP-9) in tears, thereby alleviating inflammatory responses at the ocular surface; elevated MMP-9 levels can disrupt tight junctions in the corneal epithelium and exacerbate dry eye. At the same time, diquafosol sodium can increase corneal nerve density and improve corneal sensation. Related studies suggest that diquafosol sodium increases spontaneous tear volume in rats with dry eye and reduces the number of blinks triggered by the ocular surface. Related studies have found that while diquafosol sodium activates P2Y2 receptors, it also reduces the number of neurons in the inferior trigeminal tract complex of rats with dry eye that exhibit immunoreactivity to the immediate-early gene c-Fos (cFos) and phosphorylated extracellular signal-regulated kinase (pERK), thereby alleviating ocular irritation (35). Kawahara (36) summarized evidence of increased tear secretion for 5–30 min and increased LLT for up to 60 min after instillation; Fukuoka and Arita (37) also reported an early LLT increase in healthy eyes. In our study, within-group SIT and BUT values increased, but only the SIT comparisons with standard control group at weeks 3 and 4 excluded zero after multiplicity adjustment; the corresponding BUT and LLT comparisons did not. This difference may reflect distinct assessment times and populations: prior studies examined acute effects, whereas we assessed older adults with type 2 diabetes at scheduled postoperative visits. Transient effects may have been missed. Short treatment duration, measurement variability, and limited sample size may also have contributed.

### 4.4 Perfluorohexyloctane

Median LLT in the perfluorohexyloctane group increased from 45.0 nm on postoperative day 1 to 60.5 nm at week 4. However, all adjusted LLT comparison intervals included or touched zero, so this within-group recovery does not establish superiority over standard control group or another active treatment group. Perfluorohexyloctane does not directly regulate biosecretion; rather, through its amphiphilic structure, it forms a stable perfluoroalkane layer on the tear film surface, directly supplementing and enhancing the lipid layer's anti-evaporation function. This suggests that the full formation and stabilization of the barrier require time to develop and may indirectly promote the recovery of gland function by dissolving blockages at the meibomian gland orifices and improving the fluidity of the lipid layer (38, 39). Regarding improvements in BUT, BUT in the perfluorohexyloctane group increased early throughout the observation period; this is thought to stem from the highly stable, low-surface-tension physical barrier it forms at the tear film–air interface, which effectively inhibits tear evaporation (40). Perfluorohexyloctane eye drops have extremely low surface tension, allowing them to rapidly spread across the ocular surface and interact with the lipophilic portion of the tear film, forming a protective layer at the tear film–air interface to prevent excessive tear evaporation (41). Although SIT increased within the perfluorohexyloctane group by week 4, all adjusted comparisons with standard control included or touched zero across the five outcomes. These results neither demonstrate equivalence nor validate an anti-evaporative mechanism.

### 4.5 Correlations between baseline characteristics and therapeutic responses to different medications

#### 4.5.1 Glycated hemoglobin (HbA1c) as a modifier of drug treatment efficacy

No clear HbA1c-by-treatment interaction was detected for the four continuous outcomes, including LLT (*P* = 0.448; Holm-adjusted *P* = 1.000). The provisional TFT interaction was also non-significant (*P* = 0.884), but its interpretation depends on unverified proportional-odds assumptions. Diquafosol sodium acts primarily as a P2Y2 receptor agonist, promoting mucin and aqueous secretion; its action depends on the normal function of conjunctival epithelial cells (10, 42). Impaired conjunctival function is a possible biological explanation requiring further investigation; the present study did not establish attenuated diquafosol efficacy at higher HbA1c levels. A population-based study found reduced tear MUC5AC and conjunctival goblet-cell density in diabetic patients with dry eye; HbA1c ≥ 7.0% was also associated with higher tear osmolarity and shorter fluorescein BUT (31). A 2026 cross-sectional study further linked higher HbA1c to shorter NIBUT, lower Schirmer values, and greater odds of dry eye (43). Due to chronic hyperglycemia, the ocular surface of diabetic patients is in a state of low-grade inflammation, with impaired meibomian gland function and tear secretion (44). TFT grades characterize the crystalline patterns formed after tear drying (16, 45). Electrolyte composition and the balance of salts and macromolecules influence these patterns (46, 47); TFT does not quantify mucin secretion directly. At week 4, lower grades were more frequent in the diquafosol group (Table 8), while adjusted median-change comparisons favored diquafosol over standard care at weeks 3 and 4. These findings do not establish superiority over the other active treatments or treatment-effect modification by HbA1c. Empty and sparse grade categories, particularly after stratification (Table 9), constrain interpretation of the ordinal interaction models.

#### 4.5.2 Relationship between duration of diabetes and drug efficacy across outcome measures

No clear treatment-by-diabetes-duration interaction was detected for the four continuous outcomes. The provisional TFT interaction was also non-significant [chi-square (3) = 1.851, *P* = 0.604], but model-assumption assessment was inconclusive. These findings do not establish that diabetes duration is unrelated to treatment response,particularly given the exploratory analyses and limited sample size. Although the duration of diabetes may reflect the duration of chronic metabolic exposure, there are significant individual differences among patients in terms of the time of onset of glycemic abnormalities, glycemic control, the extent of corneal nerve damage, meibomian gland function, and lacrimal reserve capacity. Therefore, stratification based solely on duration of diabetes may not be sufficient to accurately predict the response to ocular surface treatment.

#### 4.5.3 Relationship between insulin use and drug efficacy across outcome measures

Treatment-by-insulin-use interactions were not statistically significant for the four continuous outcomes. For TFT, the overall interaction was also non-significant in the fitted model [chi-square (3) = 6.043, *P* = 0.110], with incomplete proportional-odds diagnostics and sparse subgroup counts. A single unadjusted interaction coefficient cannot establish effect modification when considered selectively among multiple comparisons. Insulin use itself may not directly affect the efficacy of topical eye drops; however, further research is needed to determine whether the conditions it represents—decreased *β*-cell function, chronic low-grade inflammation, and impaired lacrimal gland function—weaken the therapeutic response to certain medications (48). Insulin use reflects systemic glycemic control strategies or the severity of diabetes rather than the state of the local ocular surface microenvironment. These differences in metabolic background may exert some influence. However, since no significant interaction between insulin use and group was observed in this study, this finding is insufficient to serve as an independent basis for guiding the selection of topical medications.

Chronic hyperglycemia can damage the sensory nerves of the cornea, reducing corneal sensitivity and affecting the reflex secretion of tears; at the same time, autonomic dysfunction can also lead to decreased secretion from the lacrimal glands. A high-glucose environment can induce the release of inflammatory factors and oxidative stress, impairing the function of corneal and conjunctival epithelial cells and goblet cells, reducing mucin secretion, and affecting tear film stability. Furthermore, diabetes can cause meibomian gland dysfunction, alter the structure of the lipid layer, and increase tear evaporation. The hyperosmolar state of tears further exacerbates ocular surface inflammation and cellular damage, creating a vicious cycle (6, 7). Meanwhile, the incision made during cataract surgery can cause acute damage to corneal nerves, making diabetic patients more prone to postoperative tear film instability, insufficient tear secretion, and delayed ocular surface healing. These pathways provide possible explanations for outcome-specific treatment responses, but the present study did not directly measure neural recovery, inflammatory activity, mucin secretion, or tear evaporation. Consequently, it cannot attribute the observed differences to a particular mechanism or establish a mechanistic hierarchy among the treatments.

In summary, all three regimens were administered on top of standard treatment (gatifloxacin eye drops three times daily for 2 weeks, combined with tobramycin-dexamethasone eye drops and pranoprofen eye drops three times daily for 4 weeks). Although the three agents differ in their mechanisms of action and clinical benefits, selected objective ocular surface parameters improved markedly during follow-up in diabetic patients after cataract surgery. The 0.05% cyclosporine group showed advantages in BUT, while the diquafosol sodium group demonstrated superiority in the Schirmer I test and tear ferning grade relative to the standard control group. However, these findings cannot establish an overall treatment hierarchy. This study suggests that anti-inflammatory treatment with 0.05% cyclosporine eye drops and tear secretion–promoting treatment with diquafosol sodium eye drops offer short-term benefits; however, an overall ranking of the treatment regimens could not be established.

## 5 Conclusion

Using a prospective, randomized, parallel-controlled design, this study compared three eye-drop regimens—0.05% cyclosporine, diquafosol sodium, and perfluorohexyloctane—for the treatment effects on postoperative ocular surface parameters in diabetic patients with cataracts, and further analyzed the potential influence of baseline factors, such as glycemic control, on treatment response. Because the study was open-label and all groups received standard postoperative care, including topical corticosteroids, the findings concern the added treatments within this regimen and do not establish their efficacy as monotherapy.

The F1-LD-F1 analysis identified treatment-by-time interactions for all five outcomes. Exploratory comparisons adjusted across 150 contrasts suggested greater improvement with cyclosporine in BUT at week 4 and with diquafosol in Schirmer I test and tear ferning grade at weeks 3 and 4 relative to standard control. The findings did not establish pairwise superiority for LLT or TMH or an overall ranking of the three treatments. These results concern short-term objective signs and require confirmation given the complete-case analysis and limitations of bootstrap uncertainty estimation.

This study still has certain limitations: First, preoperative ocular surface parameters were already abnormal, and the absence of postoperative symptom assessment precluded a formal diagnosis of dry eye according to TFOS DEWS II; thus, our findings reflect changes in objective signs rather than treatment of incident dry eye. Second, the 4-week follow-up and moderate sample size limit evaluation of long-term efficacy. Third, the trial did not assess patient treatment adherence（No adherence monitoring was performed via bottle weighing, medication logs, or questionnaires.）, which may introduce bias. Fourth, all groups received topical corticosteroids for 4 weeks; the findings therefore concern additional treatment effects within this shared regimen and do not isolate monotherapy effects or possible drug interactions. Fifth, the absence of a non-diabetic cataract control group limits generalizability to broader populations. The restriction to adults aged 50–75 years with type 2 diabetes may have introduced selection bias and limits the generalizability of our findings to younger or older patients, individuals with type 1 diabetes, and other populations. What's more, the unreported pilot data and variance assumptions prevent independent reproduction of the sample-size calculation. Cohen's reference (12) explains the interpretation of f but does not justify the study-specific estimate.

Additionally, complete-case analysis may introduce bias, and the revision-stage pairwise analyses remain exploratory. Bonferroni adjustment covered only 150 comparisons; interval validity remains limited by tied and discrete data, including a zero-width TMH interval. Within-group improvements do not establish superiority over standard control, and intervals containing zero do not establish equivalence.

Future multicenter, large-sample prospective studies with longer follow-up are needed to further verify the long-term efficacy and safety of eye drops with different mechanisms for postoperative dry eye/postoperative ocular surface imbalance in diabetic patients with cataracts. At the same time, integrating subjective symptom scores with multiple ocular surface biomarkers may further clarify the molecular mechanisms underlying the development and progression of dry eye, and help evaluate the therapeutic advantages of different drugs or combination treatment strategies across different types of tear film impairment. In addition, greater emphasis should be placed on mechanistic and clinical interventional studies to promote the development of precision treatment strategies for diabetes-related postoperative dry eye/ improve the condition of the ocular surface parameters, thereby further improving postoperative visual quality and quality of life. Given the pre-existing ocular surface dysfunction at baseline and the absence of symptom-based diagnostic criteria, the findings of this study should be interpreted as comparative effects on objective ocular surface parameters.

## Data Availability

The data supporting the findings of this study are available from the corresponding author upon reasonable request.
