# Supplementary material for: Effects of 0.05% cyclosporine, diquafosol sodium, and perfluorohexyloctane eye drops on ocular surface parameters after cataract surgery in patients with type 2 diabetes: a randomized controlled trial
Source: Front Med (Lausanne). 2026 Oct 29;13:1960289. doi: 10.3389/fmed.2026.1960289 (PMC13754669; doi:10.3389/fmed.2026.1960289)
Supplement: Supplementary file 1 [file Datasheet1.docx]

Supplementary Table 3-3. All active-treatment comparisons with Bonferroni-adjusted intervals

| **Outcome** | **Visit** | **CsA − DQS** | **CsA − PFHO** | **DQS − PFHO** |
| --- | --- | --- | --- | --- |
| LLT (nm) | D1 | -2.0 (-7.0, 12.0) | -4.0 (-7.0, 16.0) | -2.0 (-12.0, 17.0) |
|  | W1 | 0.0 (-2.5, 8.0) | 0.0 (-1.5, 17.0) | 0.0 (-1.5, 16.5) |
|  | W2 | 1.5 (-5.0, 10.0) | 3.0 (-4.5, 13.5) | 1.5 (-4.5, 11.0) |
|  | W3 | 0.5 (-4.0, 9.0) | -2.5 (-6.0, 16.0) | -3.0 (-6.0, 12.0) |
|  | W4 | 4.0 (-5.5, 13.5) | -1.0 (-12.3, 8.0) | -5.0 (-16.0, 3.8) |
| BUT (s) | D1 | -0.5 (-2.0, 1.0) | 0.5 (-2.0, 2.0) | 1.0 (0.0, 2.0) |
|  | W1 | 3.0 (0.5, 6.0) * | 4.0 (2.0, 7.0) * | 1.0 (-0.5, 3.0) |
|  | W2 | 3.0 (0.5, 5.0) * | 5.0 (2.0, 7.0) * | 2.0 (0.0, 3.5) |
|  | W3 | 1.5 (-0.5, 5.0) | 4.5 (2.0, 6.5) * | 3.0 (0.0, 4.0) |
|  | W4 | 3.0 (0.0, 4.0) | 6.0 (3.0, 7.0) * | 3.0 (0.0, 5.0) |
| TMH (mm) | D1 | 0.000 (-0.075, 0.050) | 0.000 (-0.075, 0.050) | 0.000 (-0.057, 0.050) |
|  | W1 | 0.000 (-0.040, 0.000) | 0.000 (-0.050, 0.000) | 0.000 (-0.050, 0.040) |
|  | W2 | 0.000 (-0.050, 0.080) | 0.000 (-0.050, 0.080) | 0.000 (-0.050, 0.050) |
|  | W3 | -0.035 (-0.080, 0.000) | 0.000 (-0.080, 0.000) | 0.035 (-0.065, 0.080) |
|  | W4 | -0.050 (-0.090, 0.030) | 0.000 (-0.100, 0.050) | 0.050 (-0.075, 0.090) |
| Tear ferning (grade) | D1 | 0.0 (-1.0, 1.0) | 0.0 (-1.0, 1.0) | 0.0 (-1.0, 1.0) |
|  | W1 | 1.0 (0.0, 1.0) | 0.0 (-1.0, 1.0) | -1.0 (-2.0, 1.0) |
|  | W2 | 1.0 (-0.5, 1.0) | 0.0 (-1.0, 1.0) | -1.0 (-1.5, 0.0) |
|  | W3 | 1.0 (0.0, 2.0) | 0.0 (-1.0, 1.0) | -1.0 (-2.0, 0.0) |
|  | W4 | 1.0 (0.0, 2.0) | 0.0 (-1.0, 1.0) | -1.0 (-2.0, 0.0) |
| Schirmer I (mm) | D1 | 0.0 (-2.0, 2.0) | -1.0 (-2.5, 2.0) | -1.0 (-2.5, 3.0) |
|  | W1 | -2.0 (-3.5, 0.5) | 1.0 (-3.0, 2.0) | 3.0 (-1.0, 4.0) |
|  | W2 | -1.0 (-4.0, 1.0) | 0.5 (-2.5, 3.0) | 1.5 (-1.0, 5.0) |
|  | W3 | -2.0 (-4.5, 1.0) | -0.5 (-3.0, 3.5) | 1.5 (-1.0, 5.5) |
|  | W4 | -1.0 (-5.0, 2.0) | 1.0 (-3.0, 4.0) | 2.0 (-2.0, 5.5) |

Abbreviations: LLT, lipid layer thickness; BUT, tear break-up time; TMH, tear meniscus height; CsA, 0.05% cyclosporine A; DQS, 3% diquafosol sodium; PFHO, perfluorohexyloctane; CI, confidence interval; D1, postoperative day 1; W1–W4, postoperative weeks 1–4; nm, nanometers; s, seconds; mm, millimeters. Values are differences in median individual change from baseline (first-named treatment minus second-named treatment), with Bonferroni-adjusted CIs in parentheses. * indicates that the adjusted CI excludes zero.
