# Supplementary material for: Effects of 0.05% cyclosporine, diquafosol sodium, and perfluorohexyloctane eye drops on ocular surface parameters after cataract surgery in patients with type 2 diabetes: a randomized controlled trial
Source: Front Med (Lausanne). 2026 Oct 29;13:1960289. doi: 10.3389/fmed.2026.1960289 (PMC13754669; doi:10.3389/fmed.2026.1960289)
Supplement: Supplementary file 3 [file Datasheet3.docx]

Supplementary Table 3-4B. Pointwise 95% intervals for all active-treatment comparisons

| **Outcome** | **Visit** | **CsA − DQS** | **CsA − PFHO** | **DQS − PFHO** |
| --- | --- | --- | --- | --- |
| LLT (nm) | D1 | -2.0 (-5.0, 6.5) | -4.0 (-5.0, 7.5) | -2.0 (-9.0, 9.0) |
|  | W1 | 0.0 (-2.0, 4.0) | 0.0 (0.0, 6.5) | 0.0 (0.0, 5.0) |
|  | W2 | 1.5 (-2.0, 6.0) | 3.0 (0.0, 7.5) | 1.5 (-1.0, 3.5) |
|  | W3 | 0.5 (-2.0, 6.5) | -2.5 (-3.0, 6.0) | -3.0 (-3.5, 2.0) |
|  | W4 | 4.0 (-1.5, 8.0) | -1.0 (-6.0, 3.0) | -5.0 (-9.0, -1.0) |
| BUT (s) | D1 | -0.5 (-1.0, 0.0) | 0.5 (-1.0, 2.0) | 1.0 (0.0, 2.0) |
|  | W1 | 3.0 (1.5, 4.5) | 4.0 (2.5, 5.5) | 1.0 (0.0, 2.0) |
|  | W2 | 3.0 (1.5, 4.0) | 5.0 (3.5, 6.0) | 2.0 (1.0, 3.0) |
|  | W3 | 1.5 (1.0, 3.0) | 4.5 (3.0, 6.0) | 3.0 (1.5, 3.0) |
|  | W4 | 3.0 (1.0, 4.0) | 6.0 (4.0, 6.0) | 3.0 (1.0, 4.5) |
| TMH (mm) | D1 | 0.000 (-0.050, 0.010) | 0.000 (-0.050, 0.000) | 0.000 (-0.010, 0.000) |
|  | W1 | 0.000 (-0.015, 0.000) | 0.000 (-0.010, 0.000) | 0.000 (-0.010, 0.015) |
|  | W2 | 0.000 (-0.030, 0.025) | 0.000 (-0.035, 0.025) | 0.000 (-0.035, 0.030) |
|  | W3 | -0.035 (-0.050, 0.000) | 0.000 (-0.035, 0.000) | 0.035 (-0.020, 0.050) |
|  | W4 | -0.050 (-0.080, 0.000) | 0.000 (-0.050, 0.000) | 0.050 (-0.025, 0.080) |
| Tear ferning (grade) | D1 | 0.0 (-0.5, 0.0) | 0.0 (-1.0, 0.0) | 0.0 (-1.0, 0.5) |
|  | W1 | 1.0 (0.0, 1.0) | 0.0 (-0.5, 0.0) | -1.0 (-1.5, 0.0) |
|  | W2 | 1.0 (0.0, 1.0) | 0.0 (-1.0, 1.0) | -1.0 (-1.0, 0.0) |
|  | W3 | 1.0 (0.0, 1.5) | 0.0 (-1.0, 0.5) | -1.0 (-1.5, -0.5) |
|  | W4 | 1.0 (0.0, 2.0) | 0.0 (-1.0, 1.0) | -1.0 (-2.0, 0.0) |
| Schirmer I (mm) | D1 | 0.0 (-2.0, 1.0) | -1.0 (-2.0, 0.0) | -1.0 (-2.0, 0.5) |
|  | W1 | -2.0 (-3.0, -1.0) | 1.0 (-1.5, 2.0) | 3.0 (1.0, 3.0) |
|  | W2 | -1.0 (-2.5, 0.0) | 0.5 (-1.0, 2.5) | 1.5 (0.0, 4.0) |
|  | W3 | -2.0 (-3.0, -0.5) | -0.5 (-2.0, 2.0) | 1.5 (0.0, 4.0) |
|  | W4 | -1.0 (-3.5, 1.0) | 1.0 (-1.5, 2.0) | 2.0 (-0.5, 3.5) |

Abbreviations: LLT, lipid layer thickness; BUT, tear break-up time; TMH, tear meniscus height; CsA, 0.05% cyclosporine A; DQS, 3% diquafosol sodium; PFHO, perfluorohexyloctane; CI, confidence interval; D1, postoperative day 1; W1–W4, postoperative weeks 1–4; nm, nanometers; s, seconds; mm, millimeters. Values are differences in median individual change from baseline, with unadjusted pointwise 95% bootstrap CIs in parentheses. Comparisons are active treatment minus standard control in Table 3-4A and first-named treatment minus second-named treatment in Table 3-4B.
